# Supplementary material for: Clean H2 Production by Lignin-Assisted Electrolysis in a Polymer Electrolyte Membrane Flow Reactor
Source: Materials (Basel). 2023 May 4;16(9):3525. doi: 10.3390/ma16093525 (PMC10180452; doi:10.3390/ma16093525)
Supplement: Supplementary file 1 [file materials-16-03525-s001.zip › materials-2363025-supplementary.pdf]

Supporting Information

Clean H_2 production by lignin-assisted electrolysis in a polymer electrolyte membrane flow reactor

José-Enrique Rodríguez-Fernández, María Rojo, Juan Ramón Avilés-Moreno * and Pilar Ocón

Universidad Autónoma de Madrid (UAM), Departamento de Química Física Aplicada, C/Francisco Tomás y Valiente 7, 28049 Madrid, Spain.

* Correspondence: juan.aviles@uam.es

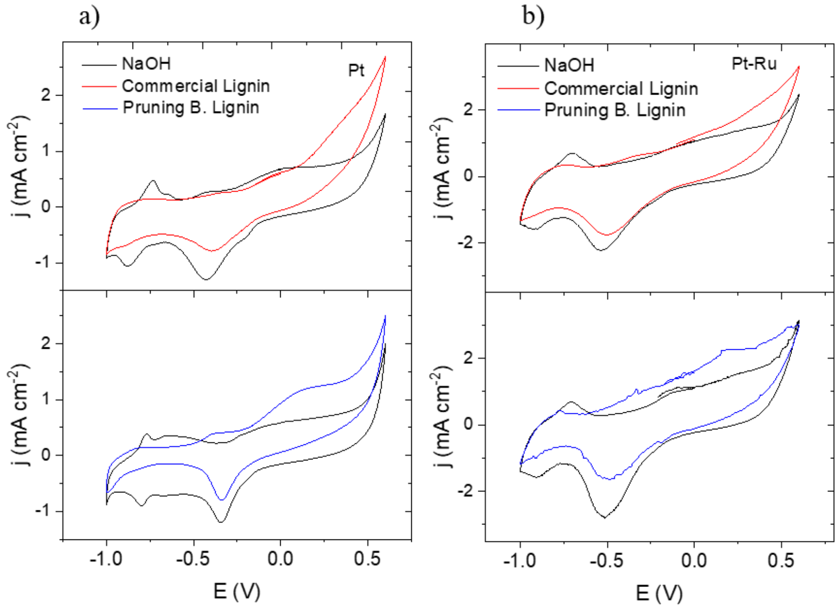

Figure S1: Cyclic Voltammetries comparison between lignin material and the NaOH solution, 50 mV s^{-1}, 60°C; a) Pt catalyst at 0.11 mg cm^{-2}, b) Pt-Ru catalyst at 0.12 mg cm^{-2}.

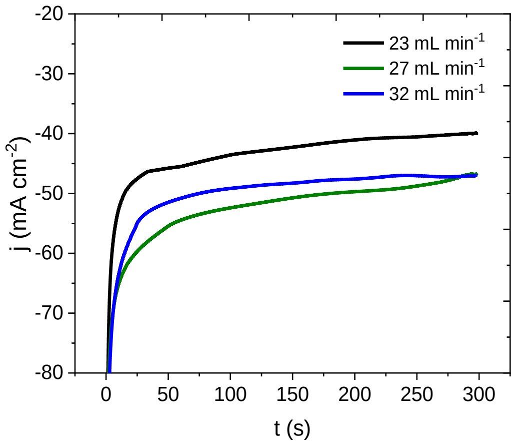

Figure S2: Chronoamperometries of Pruning B. Lignin at 6 g L^{-1}, Pt-Ru 0.09 mg cm^{-2}, -2.3 V, 300s; at different fluxes: 23, 27 and 32mL min^{-1}.

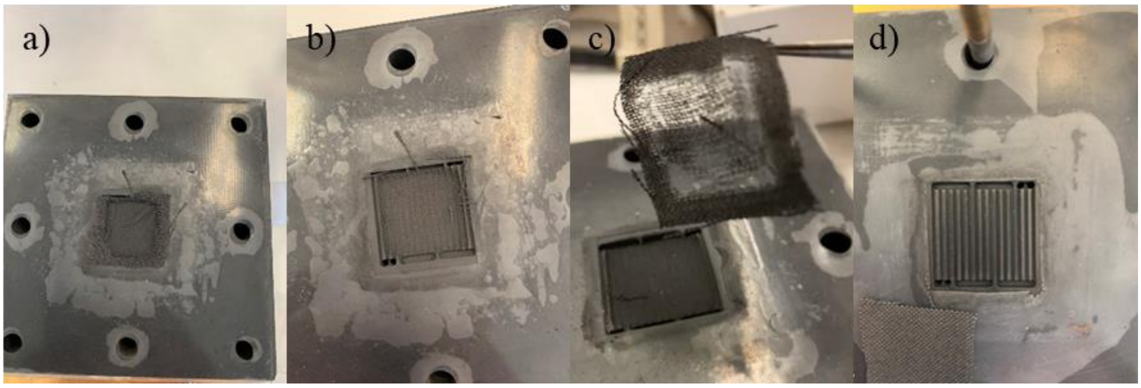

Figure S3: Graphite bipolar plates; a) separator cloth and bipolar plate from the anode degraded, b) anode plate degraded, c) separator cloth and bipolar plate from the anode degraded, d) separator cloth and bipolar plate from the cathode.

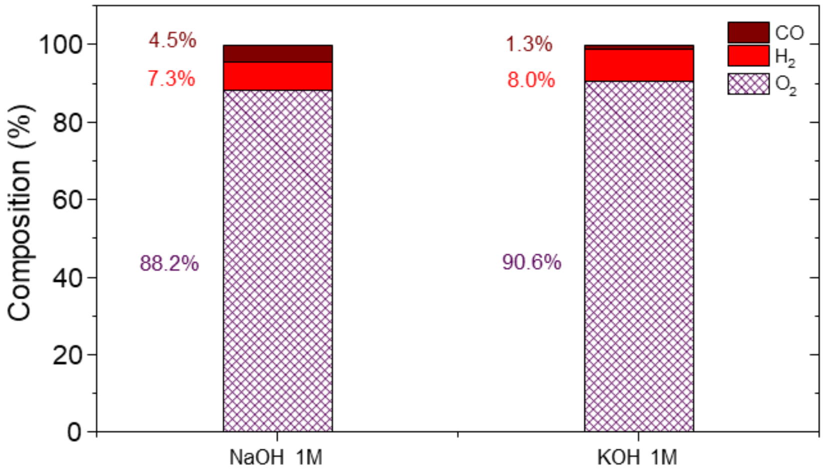

Figure S4: Gas composition with different electrolytes, -100 mA cm^{-2}, Pt at 0.08 mg cm^{-2}. 2 measurements for each quantification. Standard deviations are not showed in the graph for clarity, average obtained values are below 10% of error in the quantification of CO_2, CO, H_2 and O_2.

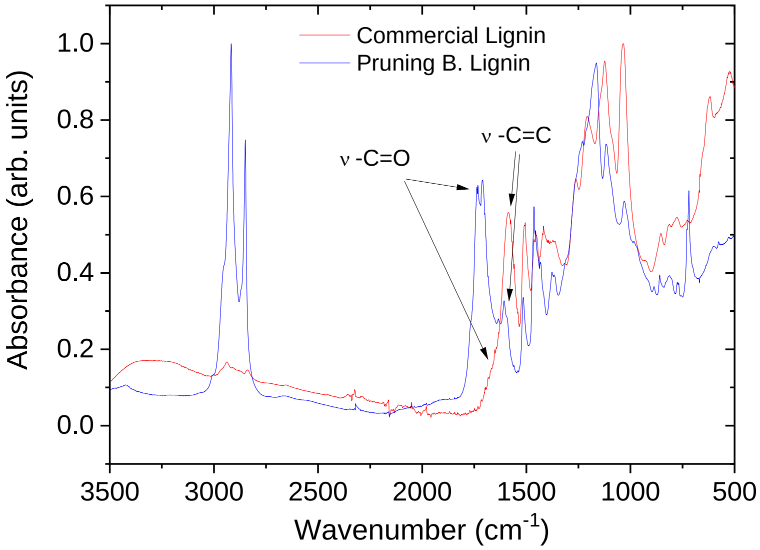

Figure S5: Commercial and Pruning B. Lignin IR spectra of dry products.
